# Low Cholesterol Level Linked to Reduced Semantic Fluency Performance and Reduced Gray Matter Volume in the Medial Temporal Lobe

**DOI:** 10.3389/fnagi.2020.00057

**Published:** 2020-03-31

**Authors:** Fan Nils Yang, Macdonell Stanford, Xiong Jiang

**Affiliations:** ^1^Department of Neuroscience, Georgetown University Medical Center, Washington, DC, United States; ^2^School of Medicine, Georgetown University Medical Center, Washington, DC, United States

**Keywords:** total cholesterol, gray matter volume, cognitive function, semantic fluency, medial temporal lobe

## Abstract

Hyperlipidemia has been proposed as a risk factor of dementia and cognitive decline. However, the findings of the relationship between cholesterol level and cognitive/brain function have been inconsistent. Here, using a well-controlled sample from the Parkinson’s Progression Markers Initiative (PPMI), we investigated the probable non-linear relationship between plasma total cholesterol (TC) level, gray matter volume (GMv), and cognitive performance in 117 non-demented subjects (mean age, 61.5 ± 8.9 years), including 67 Parkinson’s disease (PD) patients and 50 demographically matched controls. A quadratic relationship between semantic fluency (SF) performance and TC levels was identified. Within the subjects with a desirable TC level (TC < 200 mg/dl), low TC (lTC) levels were associated with reduced SF performance, as well as reduced GMv in three medial temporal regions [including bilateral anterior hippocampus (HIP)]. In contrast, no significant relationship between TC and cognition performance/GMv was found in individuals with a high cholesterol level (i.e., TC ≥ 200 mg/dl). Further region of interest (ROI)-based analysis showed that individuals with TC levels ranging from 100 to 160 mg/dl had the lowest GMv in the medial temporal regions. These findings suggest that low-normal TC level may be associated with reduced cognitive function and brain atrophy in regions implicated in neurodegenerative diseases, adding to a growing body of literature supporting a probable non-linear relationship between cholesterol level and brain health. However, this finding needs to be verified with other large public cohort data that do not include PD patients.

## Introduction

Dyslipidemia, especially hyperlipidemia, is highly prevalent in adults worldwide (World Health Organization, 2014). In the United States, the prevalence of hyperlipidemia is even more alarming: 39.7% of adults have borderline high (>200 mg/dl) or high (>240 mg/dl) total cholesterol (TC) level (Mackey et al., 2017). This is worrisome as hyperlipidemia [high TC (hTC)] has been shown to be a strong predictor of cardiovascular disease (CVD) and is a core metric in most CVD risk calculators. In addition, studies have suggested that hTC in midlife is also a potential risk factor for dementia in late life (Shepardson et al., 2011; Anstey et al., 2017).

However, while the relationship between cholesterol levels [TC and its subcomponents: low-density lipoprotein (LDL), high-density lipoprotein (HDL), and triglycerides (TGs)] and cognitive functions has been extensively studied, the findings have been largely inconsistent. For example, while some studies have found higher TC and/or higher LDL was associated with poorer cognitive performance (Yaffe et al., 2002; Carlsson et al., 2009; Sparks et al., 2010; Meusel et al., 2017) or a higher risk of dementia such as Alzheimer’s disease (AD) (Kivipelto et al., 2005; Hayden et al., 2006; Solomon et al., 2009), others have found no such effects (Mielke et al., 2010), or even the opposite pattern, i.e., higher TC and/or higher LDL levels were associated with better cognitive performance (Elias et al., 2005; West et al., 2008; van den Kommer et al., 2012; Aine et al., 2014; Lv et al., 2016) or lower risk of dementia/cognitive decline (Mielke et al., 2005; Reitz et al., 2005, 2010; van den Kommer et al., 2009). In addition, although many studies have suggested that HDL is positively correlated with cognitive functions (van den Kommer et al., 2012; Elias et al., 2014) and high HDL level is associated with reduced risk of future dementia (Reitz et al., 2010; Ancelin et al., 2013), exceptions can be found (Ancelin et al., 2014), in addition to null findings in many other studies. Many factors might have contributed to the inconsistence, such as difference in study samples (e.g., age, education, race, etc.) and neuropsychological tests in different studies. One potentially important factor is that there may be a non-linear relationship (e.g., a quadratic function) between cholesterol levels and cognitive functions, as suggested by several recent studies (Wendell et al., 2014, 2016; Lu et al., 2017; Marcum et al., 2018).

Brain imaging studies of cholesterol level have been focusing on cardiovascular risk/CVD. Among the few studies that have investigated the relationship between cholesterol level and brain structure/function, one of the most consistent findings is that higher HDL levels have been linked to less brain atrophy in middle-aged to older adults. With a group of 183 healthy adults (mean age 58.4 years), HDL levels were found to be positively correlated with gray matter volume (GMv) in bilateral temporal poles, middle temporal gyri, temporo-occipital gyri, and left superior temporal gyrus and parahippocampal region (Ward et al., 2010). In another study involving healthy older controls and individuals with mild cognitive impairment (MCI) or AD, low HDL was associated with low hippocampal volume (Wolf et al., 2004). In a very recent study with older adults (75 years old or older) who had subjective memory complaints, HDL was positively correlated with memory performance and gyrification indices of bilateral insular and frontal opercular cortices (Kinno et al., 2019). In a large longitudinal cohort study, higher HDL levels were associated with less steep GMv decline in the entorhinal cortex and parahippocampal gyrus, as well as a lower risk of future cognitive impairment (Armstrong et al., 2019).

By contrast, the findings of the relationship between brain structure/function and LDL or TC level have been less consistent. For instance, with 82 cognitively normal older adults about the same age (77.7–78.9 years old), Whalley et al. (2003) revealed that total GMv negatively correlated with LDL and TC (Whalley et al., 2003). Qiu et al. (2012) found a similar effect, but only in men and only in the hippocampus (HIP) and the entorhinal cortex (Qiu et al., 2012). Using fluorodeoxyglucose (FDG)-PET, Reiman et al. (2010) found that higher TC in cognitively normal adults (47–68 years old) was associated with hypometabolism in the precuneus and parietotemporal and prefrontal regions, all of which are known to be preferentially affected in AD (Herholz et al., 2002). Using diffusion tensor imaging (DTI) techniques, Williams et al. (2013) provided evidence suggesting that a higher LDL was associated with reduced white matter integrity in the right frontal and temporal regions, the superior longitudinal fasciculus, and the internal/external capsules (Williams et al., 2013). However, using resting-state functional MRI (fMRI) techniques, Zhang et al. (2016) found that higher TC was associated with both increased connectivity in the default mode network and reduced connectivity in the salience network, suggesting a more complicated picture (Zhang et al., 2016). Furthermore, in several recent studies, higher LDL or TC levels were linked to thicker cortical thickness (Leritz et al., 2011; Coutinho et al., 2017), increased GMv in the frontal cortex and the posterior cingulate cortex (PCC) (but only in hypertensive adults) (Chung et al., 2018), and white matter integrity (Aine et al., 2014), suggesting an opposite pattern, i.e., high cholesterol level could be potentially beneficial in middle-aged to older adults.

Taken together, these previous studies suggest that the relationship between cholesterol level and brain/cognitive function warrants further research. In the present study, we investigated the probable non-linear relationship between plasma TC level, GMv, and cognitive performance in a well-controlled and well-matched sample from the Parkinson’s Progression Markers Initiative (PPMI) cohort. Specifically, we tested two hypotheses derived from previous studies. First, we predicted a quadratic effect between TC and cognitive performance, i.e., an inverted U-shape, with both low and hTC associated with lower cognitive performance than mid-range TC. Second, accordingly, we predicted that both low and hTC associated with reduced GMv. These two hypotheses were examined in the whole subject group as well as the subgroups that were defined based on TC levels.

## Results

All data were downloaded from the PPMI website, and a total of 117 participants were included in the present study [44 female, 73 male; 67 Parkinson’s disease (PD) patients, 50 control participants]. In all data analyses (unless otherwise specified), participants were divided into two groups: lTC group, TC < 200 mg/dl (*n* = 69); and hTC group, TC ≥ 200 mg/dl (*n* = 48).

The demographic data of the hTC group and the lTC group are shown in Table 1. There were no significant differences in age, years of education, the percentage taking medicine for lowering TC, the percentage of PD patients, or Geriatric Depression Scale scores. The percentage of female was lower in the lTC than in the hTC group, but the significance did not survive after being corrected for multi-comparison. As expected, there were significant differences in TC between groups (*p* < 0.001). Similar group differences were found in LDL (*p* < 0.001) and HDL (*p* < 0.01) but not in TG (Table 1).

**TABLE 1:** Demographic information.

**Groups**	**lTC (*n* = 69)**	**hTC (*n* = 48)**	**Group difference**
Age	61.4 (9.7)	61.6 (7.7)	n.s.
Education	15.1 (3.0)	15.3 (2.9)	n.s.
Gender (female%)^*a*^	29%	50%	*p* = 0.021
Medicine (taking med%)^ a^	34.8%	22.9%	n.s.
PD/control (PD%)^ a^	40.6%	45.8%	n.s.
APOE ε4 carrier (%)^*a*^	36.2%	25%	n.s.
Geriatric Depression Scale	5 (1.2)	5.1 (0.9)	n.s.
Total cholesterol	164.0 (24.6)	222.5 (16.7)	*p* < 10^–18^
Low-density lipoprotein	88.7 (22.2)	133.4 (21.4)	*p* = 0.008
High-density lipoprotein	53.6 (18.4)	63.9 (22.7)	*p* < 10^–26^
Triglycerides	106.7 (50.5)	107.7 (54.2)	n.s.

*Data are presented as mean (standard deviation). Subjects were divided into two groups: low total cholesterol (lTC) group, subjects with desirable TC range (TC < 200 mg/dl), and high TC (hTC) group, subjects with borderline-high or high TC level (TC ≥ 200 mg/dl). ^*a*^Contingency χ^2^ test. PD, Parkinson’s disease; n.s. not significant.*

**FIGURE 1:**
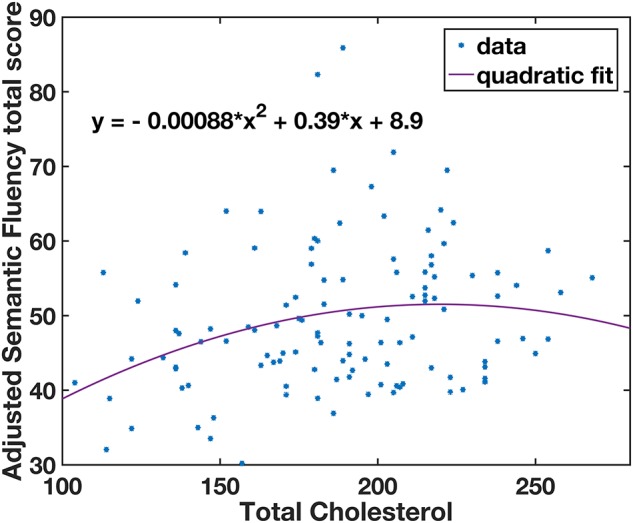
A quadratic relationship between semantic fluency (SF) scores and total cholesterol (TC) levels. The adjusted SF total scores were calculated by regressing out the covariates (age, gender, education years, Geriatric Depression Scale, patient category, taking medicine for lowering cholesterol, and APOE ε4 carrier; see section “Materials and Methods”). *Individual subjects’ data.

**FIGURE 2:**
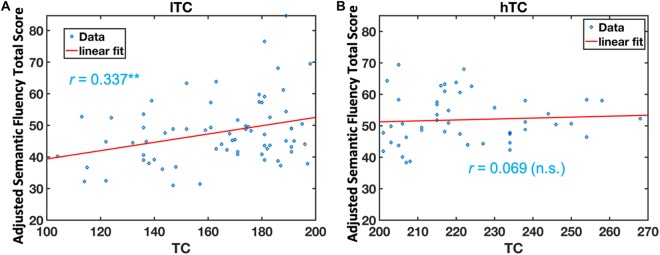
The relationship between semantic fluency (SF) scores and total cholesterol (TC) in the low TC (lTC) and the high TC (hTC) group, separately. The adjusted SF total scores were significantly correlated with TC levels in the lTC group (*r* = 0.337, *p* < 0.01) **(A)**, but not in the hTC group (*r* = 0.069, *p* > 0.05). **(B)** lTC group, participants with TC < 200 mg/dl; hTC group, participants with TC ≥ 200 mg/dl.

Multivariate analysis of covariance (MANCOVA) analysis revealed that there was no significant group difference in all cognitive measurements between lTC and hTC (at least *p* > 0.05; Table 2). However, a significant effect was found between TC and semantic fluency (SF) total score [*F*(1,107) = 6.262, *p* = 0.014]. To further test whether there is a quadratic effect between TC and SF total score, a quadratic regression analysis was performed. Indeed, a significant quadratic effect was found between TC and SF total score (*p* = 0.013; Figure 1). Additional partial Pearson correlation analyses revealed that in the lTC group, TC was positively correlated with SF total score (*r* = 0.337, *p* < 0.01; Figure 2A), but not in the hTC group (*p* > 0.05; Figure 2B), and similar results were obtained when HDL was controlled.

**TABLE 2:** Neuropsychological test scores.

**Cognitive domains**	**Tasks**	**lTC (*n* = 69)**	**hTC (*n* = 48)**	**Group Difference**
Global	MoCA	27.6(1.9)	27.6(2.1)	n.s.
Visuospatial	Line Orientation Score	13.1(2.2)	13.1(1.6)	n.s.
Executive abilities—working memory	Letter Number Sequencing Raw Score	10.6(2.7)	10.7(2.6)	n.s.
	Semantic Fluency Total Score	47.8(11.3)	51.9(10.0)	n.s.
Processing speed—attention	Symbol Digit Modalities Score	43.7(11.3)	43.6(10.8)	n.s.
Memory	HVLT-R Immediate Recall	24.9(4.2)	25.3(4.2)	n.s.
	HVLT-R Delayed Recall	8.3(2.6)	8.6(2.3)	n.s.
	HVLT-R Retention	0.8(0.2)	0.9(0.2)	n.s.
	HVLT-R Discrimination Recognition	9.8(2.4)	9.6(3.1)	n.s.

*Data are presented as mean (standard deviation). Low total cholesterol (lTC), subjects with TC < 200 mg/dl; high TC (hTC), subjects with TC ≥ 200 mg/dl. HVLT-R, Hopkins Verbal Learning Test-Revised; MoCA, the Montreal Cognitive Assessment; n.s., not significant.*

**FIGURE 3:**
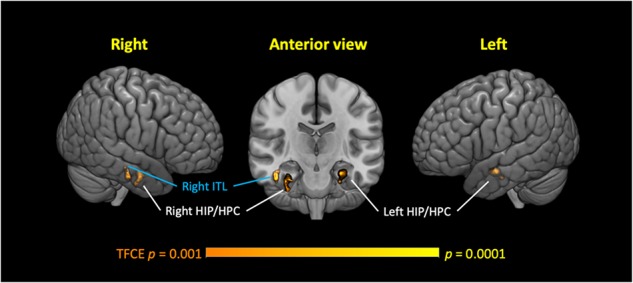
Low-normal total cholesterol (TC) levels correlated with reduced gray matter volume (GMv) in three medial temporal regions in the low TC (lTC) group. Three significant clusters were identified, including the bilateral anterior hippocampus (HIP)/parahippocampal cortex (HPC), and the right inferior temporal lobe (ITL). Age, gender, education years, Geriatric Depression Scale, patient category, whether or not taking medicine for lowering cholesterol, apolipoprotein (APO)E ε4 carrier status, and brain size were controlled (see section “Materials and Methods”). Thresholds, *p* < 0.001 (Threshold-Free Cluster Enhancement, uncorrected, voxel-wise), *p* < 0.05 [family-wise error rate (FWE) corrected at cluster level], and cluster size (≥50 voxels). TFCE, threshold-free cluster enhancement.

Voxel-based morphometry (VBM) analyses of GMv were conducted separately for the lTC and hTC groups, using the TC as the covariate, after controlling for other potential confounding factors (see section “Materials and Methods”). The VBM analyses revealed that in the lTC group, TC was positively correlated with GMv in three medial temporal regions, including the bilateral anterior HIP/parahippocampal cortex (PHC), and right inferior temporal lobe (ITL) [*p* < 0.05 cluster-wise family-wise error rate (FWE) corrected, Figure 3; see Supplementary Table S3 for coordinates of the peak voxel for these three clusters]. In the hTC group, however, no voxels survived at the threshold of *p* < 0.001 [threshold-free cluster enhancement (TFCE) uncorrected].

**FIGURE 4:**
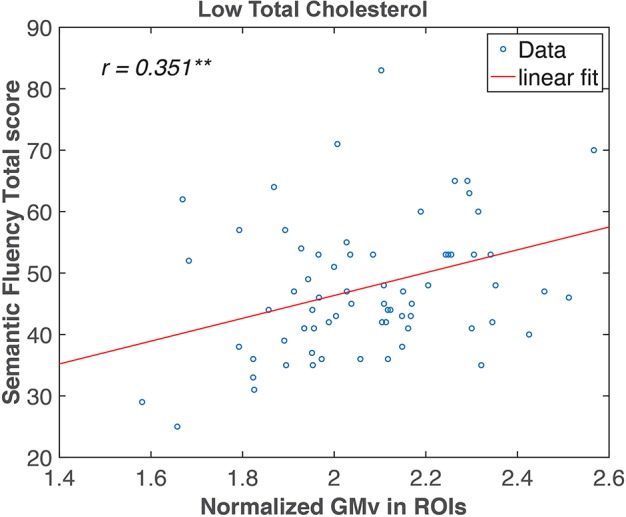
Semantic fluency (SF) total score correlated with gray matter volume (GMv) in the medial temporal regions in the low total cholesterol (lTC) group. Pearson correlation analysis revealed a significant correlation between the normalized total GMv of the three clusters identified in Figure 3 and SF in the lTC group (*r* = 0.351, *p* < 0.01). ROI, region of interest. ** *p* < 0.01.

**FIGURE 5:**
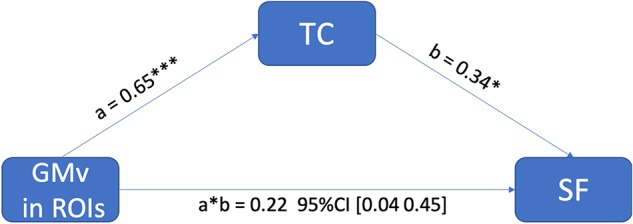
The positive effect of gray matter volume (GMv) in medial temporal regions of interest (ROIs) on semantic fluency (SF) performance was mediated through total cholesterol (TC). The standardized indirect effect from GMv to SF was significant, a * b = 0.22, 95% CI (0.04–0.45) in 5000 bootstrapped samples, and the direct effect was not significant (*p* = 0.90). Note: ***p < 0.001; *p < 0.05.

In the region of interest (ROI)-based analysis, the GMv of the three clusters identified in Figure 3 was extracted, summed, and normalized with total intracranial volume (TIV). The total normalized GMv of three clusters was positively correlated with SF total score (*r* = 0.351, *p* < 0.01; Figure 4). Mediation analyses among total GMv of the three clusters, SF total score, and TC were conducted to disentangle the relationship among these three variables. A significant indirect effect of GMv on SF total score through TC was found (standardized indirect effect = 0.22, 95% CI [0.04–0.45] in 5000 bootstrap samples; Figure 5).

**FIGURE 6:**
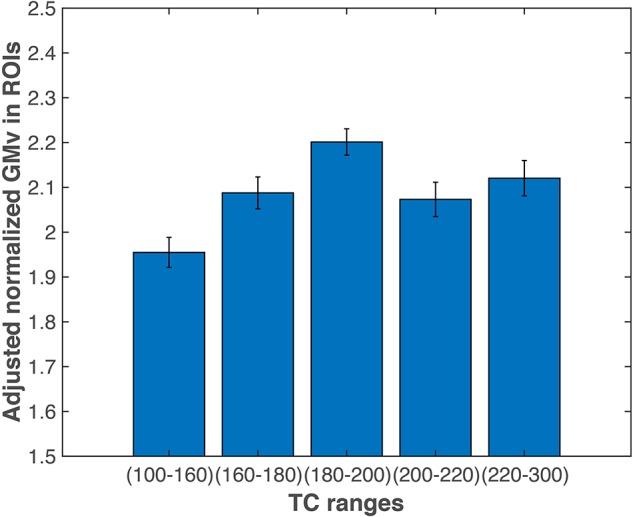
Gray matter volume (GMv) of the medial temporal regions of interest (ROIs). Participants with total cholesterol (TC) level lower than 160 mg/dl had significantly lower total GMv in the three medial temporal ROIs (see Figure 3) than the other four groups (at least *p* < 0.05). Error bars represent SEM. GMv, gray matter volume; TC, total cholesterol.

In an additional analysis to further investigate the relationship between TC and GMv, we divided all the subjects into five groups based on TC levels (Figure 6; also see section “Materials and Methods”). ANCOVA revealed a significant group effect for the total GMv of the three ROIs [*F*(4,112) = 6.87, *p* < 0.0001]. *Post hoc* analyses showed that groups with TC level below 160 has significantly lower GMv than all other four groups (at least *p* < 0.05; Figure 6).

## Discussion

In the present study, using a well-matched and well-controlled sample from the PPMI dataset, we provided evidence suggesting a non-linear relationship between TC level and cognitive performance/GMv. Specifically, the data suggested that low-normal TC (<160 mg/dl) was associated with reduced performance on SF task and reduced GMv in three medial temporal regions, including bilateral anterior HIP. Further mediation analysis suggested that in these participants, the effects of GMv on reduced SF performance were mediated through TC.

The non-linear (inverted U-shaped) relationship between TC and cognitive performance in the present study is consistent with findings from several recent studies (Wendell et al., 2014, 2016; Lu et al., 2017; Marcum et al., 2018). Using data from the Baltimore Longitudinal Study of Aging, Wendell et al. (2014) identified two opposite non-linear relationships between cholesterol levels and performance on several neuropsychological tests (including letter verbal fluency) in healthy older adults (age 54–83 years); that is, a U-shape in those 70 or older (i.e., worst performance with midrange cholesterol level) and an inverted U-shape in those younger than 70 (i.e., best performance with midrange cholesterol level); a similar non-linear relationship was also found with LDL (Wendell et al., 2016). In a cohort study in China (age 50–65 years), Lu et al. (2017) identified an inverted U-shape relationship between TC/LDL and neuropsychological test scores in men versus a U-shaped relationship between HDL and neuropsychological test scores in women (Lu et al., 2017). These studies suggest that the relationship between cholesterol levels and cognitive performance is likely non-linear, and the precise relationship might depend on age, gender, and probably other demographic factors as well. Highly relevant to the present study, a large cohort study [Adult Changes in Thought (ACT) Study, *n* = 6821] revealed older adults (60–80 years old) with low (120 mg/dl) and high (210 mg/dl) non−HDL cholesterol levels had modestly higher risk of AD than those with intermediate (160 mg/dl) level (Marcum et al., 2018). Compared to those previous studies with larger samples, the present study does not have enough power to investigate the probable different roles of age and gender, but the results are in a general agreement with findings from these recent studies (Wendell et al., 2014, 2016; Marcum et al., 2018), especially since the majority of the subjects in the PPMI dataset were 70 or younger (similar results were observed after excluding subjects older than 70).

The present study extended the abovementioned results of non-linear relationship to a simple yet widely used neuropsychological test for the detection of dementia: SF (more specifically, animal fluency) test. SF performance has been linked to medial temporal lobe (MTL) and anterior temporal cortex (Pelletier et al., 2017), and there is a large body of evidence supporting a diagnostic role of SF test in dementia (Sebaldt et al., 2009; Sutin et al., 2019), including AD (Henry et al., 2004). However, few studies have identified a significant relationship between cholesterol level and SF performance. One study suggests that higher levels of non-HDL cholesterol are associated with poorer SF performance (Takeda et al., 2017). Another study reveals evidence suggesting higher levels of midlife TC levels might be linked to poorer SF performance and episodic memory in late life (Solomon et al., 2009). The results from these previous studies are different (even opposite) from the present study; this could be due to multiple factors, including difference in demographics, i.e., Hispanics (Takeda et al., 2017) versus mainly Caucasians in the present study. Future studies are needed to consolidate the inconsistence. However, the present study is in line with several previous studies using letter verbal fluency and with sample sizes much larger than the present study. For instance, Elias et al. (2005) suggested that word verbal fluency (not SF) is positively correlated with TC (Elias et al., 2005), and Ylilauri et al. (2017) provided evidence suggesting that moderate egg intake is associated with better cognitive performance (including word verbal fluency), which implicates that maintaining a certain cholesterol level might be beneficial to maintain cognitive function in older adults since eggs are a major source of dietary cholesterol (Ylilauri et al., 2017). Taken together, it is possible that both low and high cholesterol levels are associated with poor cognitive performance and increased risk of cognitive decline, and low cholesterol level might be associated with early pathological changes similar to those seen in AD and dementia (especially given the diagnostic role of SF in dementia). This hypothesis is further supported by the findings of reduced GMv in medial temporal regions in adults with lTC (TC < 160 mg/dl) in the present study (see below).

Underlying neural mechanism of the relationship between cholesterol levels and cognitive performance/decline has not been well established. While studies have generally agreed that high HDL levels might be beneficial to brain health (Wolf et al., 2004; Ward et al., 2010; Armstrong et al., 2019; Kinno et al., 2019), the relationship between hTC/LDL levels and brain function/structure remains controversial; both negative (Whalley et al., 2003; Reiman et al., 2010; Qiu et al., 2012; Williams et al., 2013) and positive (Leritz et al., 2011; Aine et al., 2014; Coutinho et al., 2017; Chung et al., 2018) relationships have been identified, even in the same sample (Zhang et al., 2016). In the present study, we provided evidence suggesting low-normal TC (TC < 160 mg/dl) is associated with reduced GMv in medial temporal regions (including bilateral anterior HIP). Given the fact that GMv loss in medial temporal regions and HIP has been repeatedly reported in prodromal AD (Bell-McGinty et al., 2005; Whitwell et al., 2007) and hippocampal volume in midlife has been proposed as a strong risk predictor of AD (Coupé et al., 2019), the link between low-normal TC and GMv loss in these regions in the present study is worrisome, as it is possible that low-normal TC might potentially predispose individuals to AD or dementia. Indeed, a recent study has demonstrated a U-shaped association between AD and non-HDL cholesterol levels in old adults (60–79 years old) (Marcum et al., 2018). Future longitudinal studies are needed to investigate the relationship between low-normal TC range, brain health (such as GMv in MTL), and risk of future dementia such as AD.

Nonetheless, our findings supported the assumption from Wendell et al. (2016) that there might be distinct neural correlates underlying the detrimental effect of low versus hTC on cognitive functions (Wendell et al., 2016). That is, lTC was cognitively detrimental because it has an adverse effect on brain microstructure and function, while the association between hTC and poor cognition might be mediated through other cardiovascular risk factors comorbid with hTC. In line with this hypothesis, the present study demonstrated that only in participants with desirable TC levels (i.e., TC < 200 mg/dl), TC is positively correlated with GMv in the medial temporal regions (including bilateral HIP), and the individuals with TC less than 160 mg/dl have the lowest GMv in these ROIs than others (Figure 6). The lack of significant correlation between TC and cognitive performance and GMv in the hTC group (i.e., TC > 200 mg/dl) might be due to a small sample size and insensitive measurement (i.e., SF and GMv might be insensitive to detect cognitive decline/brain injury associated with hTC), among other potential factors. Future studies utilizing multi-model neuroimaging methods and more comprehensive neuropsychological battery might help to gain a better understanding of how TC and its subcomponents affect the human brain/cognitive function.

There are several limitations of the present study. First, the PPMI cohort used in the present study does not include old-old participants (i.e., age ≥ 80). Therefore, our results cannot be generalized to these old-old population. Previous studies have shown that hTC might be beneficial to this population (Lv et al., 2016; Wendell et al., 2016). However, this association might simply reflect selective survival, as a recent meta-analysis failed to find any relationship between late-life TC and dementia. Future studies are needed to fill this gap regarding the relationship between late-life TC and cognitive decline/dementia. Second, we did not find any significant neural correlates in participates with higher-than-normal TC. This could be due to the fact that the present study did not have enough power or the neural mechanism between TC and cognitive/brain function in the hTC range might be different (i.e., different neuropsychological tests and/or brain imaging techniques are needed). Future studies might need to replicate our study with a larger sample size and with more comprehensive neuropsychological tests and/or multimodal neuroimaging techniques. Third, the apolipoprotein E (ApoE) is the major transporter of cholesterol in the brain and the major Apo regulating lipoprotein metabolism. ApoE is encoded by the polymorphic APOE gene. APOE ε4 is the gene variant that is associated with increased risk of late-onset sporadic AD and may potentially predispose carriers to hypercholesterolemia. In the present study, approximately one-third of PD patients and controls were APOE ε4 carriers, with a prevalence slightly higher than the general population in the United States (approximately 15%). However, the relatively small sample size limited our capability of detecting a significant effect of APOE ε4 status on TC, LDL, HDL, or TG levels (at least *p* > 0.5) (this was further complicated by the fact that nearly 30% of participants were taking medication to control blood cholesterol levels; Table 1). Therefore, future longitudinal studies with a large sample size might be necessary to fully explore the potential interactions between APOE ε4, age, and cholesterol levels on brain structure and function (especially in the context of AD risk). Fourth, while the results from the present study and several previous studies suggest that it might be beneficial to maintain certain cholesterol levels (i.e., TC > 160 mg/dl) in middle-aged to older adults, this conclusion should be taken with caution as individuals who are actively enrolled in a study are likely to be on top of their healthcare and might have reduced cardiovascular issues compared to the general population. In addition, the results cannot be generalized to the old-old population (i.e., 80 or older) as the participants were relatively young in the PPMI cohort (with an average around 61.5 years old). Therefore, the comprehensive relationship between cholesterol levels (including each of the subcomponents), cognitive function, risk of dementia, and brain health/function remains an open question that needs to be addressed in future studies.

In summary, our findings suggested that adults with low-normal range TC are likely to have smaller GMv in the medial temporal regions that have been shown to be preferentially affected in AD, along with a reduced performance in SF that has been used as a tool in dementia diagnosis. In addition, the mediation analysis suggests that TC mediates the relationship between brain atrophy and SF performance in those adults. Taken together, the present study might have important clinical implications that the optimal TC range might be somewhere between 160 and 200 mg/dl, so cautions should be taken to control the potential adverse relationship between lTC and GMv, while being aggressive to control the cardiovascular risk associated with hTC. Future studies and replications are warranted to identify the causal relationships among TC, brain health (including brain atrophy), and cognitive performance.

## Materials and Methods

### Participants

The PPMI study is a multicenter PD study designed to identify PD-related biomarkers. Detailed information about the study design can be found elsewhere (Marek et al., 2011) and at the website^1^. This study was approved by the institutional review boards of 21 research sites located in Australia, Europe, and the United States (see https://www.ppmi-info.org/about-ppmi/ppmi-clinical-sites/for a complete list). Written informed consent from every participant was obtained prior to study enrollment. Per statement on the PPMI website, all methods were performed in accordance with the relevant guidelines and regulations. The detailed info is too long to be included in a paper but can be found at the website^2^, especially under Study Design^3^. All participants were evaluated by comprehensive clinical (motor, neuropsychological, and cognitive) and imaging [dopamine transporter (DAT) imaging] assessments and bio-sampling at screening, baseline, and follow-up sessions. PD diagnosis within 2 years and DAT deficit were required for PD participants’ eligibility at screening. Healthy controls were matched to PD patients and had normal cognition, no neurological disorders, and no first-degree family member with PD. The present study used a subset of the PPMI participants (*n* = 125, 45 female, 80 male) with the following criteria: available magnetization-prepared rapid gradient-echo (MPRAGE) images and TC measurements. PD patients and controls were collapsed together (no significant difference between PD and controls were found in TC, and similar but less significant results were observed with only PD patients or controls, see Supplementary Material), and disease status was included as a covariate in the data analysis. Of the 125 participants, six participants with geriatric depression scale higher than 8 were excluded, and two additional participants with extreme TC level (>300 or <100) were also excluded. The demographic information of the remaining 117 participants (44 female, 73 male; 67 PD patients, 50 control participants) is shown in Table 1, and the MR images from the 117 participants passed the quality control following the standard procedure and the default criteria in the software packages (see below).

### Neuropsychological Tests

Per PPMI website^1^, neuropsychological tests of the following five domains were administered: Montreal Cognitive Assessment (MoCA)-global cognitive function; SF and Letter–Number Sequencing-working memory and executive function; Symbol–Digit Modalities Test-speed of information processing; Hopkins Verbal Learning Test-Revised (HVLT-R)-learning and memory; and Benton Judgment of Line Orientation 15-item (split-half)-visuospatial function (Table 2). The neuropsychological tests used in the PPMI study were conducted in a strictly controlled environment according to the general test guidelines for PPMI (see http://www.ppmi-info.org/wp-content/uploads/2010/04/PPMI-General-Operations-Manual.pdf Section 6 for detailed information). Specifically, all the tests happened in a quiet room, with a properly trained examiner and an examinee seated on opposite sides of a table.

### Cholesterol Measurements (Plasma)

Blood sample collection and processing were described in detail in the PPMI protocol manual^1^. Blood samples were obtained during 8:00–10:00 a.m. after an overnight fast. Plasma concentrations of TC, TG, LDL, and HDL were measured. In all data analyses (unless otherwise specified), participants were divided into two groups: lTC group, TC < 200 mg/dl (*n* = 69); and hTC group, TC ≥ 200 mg/dl (*n* = 48). See Table 1 for demographic information of each group. In addition, the demographics of PD patients versus controls are shown in Supplementary Table S1. The neuropsychological test scores of four subgroups, PD with lTC level (lTC PD), PD with hTC level (hTC PD), controls with lTC (lTC Controls), and controls with hTC level (hTC Controls), are shown in Supplementary Table S2.

### MRI Acquisition and Preprocessing

High-resolution T1-weighted images were acquired with 3D-MPRAGE at 1 mm^3^ × 1 mm^3^ × 1.5 mm^3^ (or 1 mm^3^ × 1 mm^3^ × 1.2 mm^3^) resolution. Detailed acquisition parameters can be found at the PPMI website^4^. The software package SPM12^5^ and the toolbox Computational Anatomy Toolbox (CAT, version 12.5)^6^ were used for preprocessing and VBM analyses. Default processing pipeline settings of the CAT were applied, including tissue segmentation, normalization, quality control, and smoothed with an 8-mm full-width at half-maximum (FWHM) Gaussian kernel.

### Statistical Analysis

The statistical analyses were performed using standard methods in SPSS 25.0 (Chicago, IL, United States), MATLAB 2018a (Math Works, Natick, MA, United States). All statistical analyses were two-tailed. Because of the assumption of a non-linear relationship between TC and cognitive/brain function (which was supported by a quadratic function between TC and SF test scores, see Figure 1), we divided the participants into two groups, a lTC group, i.e., with a desirable TC level (TC < 200 mg/dl) and a hTC group, i.e., with a borderline high or hTC level (TC ≥ 200 mg/dl). Unless otherwise explicitly specified, all analyses were conducted independently for each of the two groups.

Contingency χ^2^ tests and two-sample *t*-tests were used to examine probable group differences in demographics between the lTC and the hTC groups (Table 1).

MANCOVA was used to test the relationship between cognitive measurements and TC in the entire participant group. In this analysis, nine cognitive measurements were defined as dependent variables: MoCA Score, Line Orientation Score, Letter Number Sequencing Raw Score, SF Total Score (the total number of animal, vegetable, and fruit words), Symbol Digit Modalities Score, HVLT-R Immediate Recall, HVLT-R Delayed Recall, HVLT-R Retention, and HVLT-R Discrimination Recognition. TC groups [defined as a dichotomous variable: hTC (1), lTC (0)] were entered in the model as fixed factors. Independent variables were TC and seven covariates: age, gender, education years, Geriatric Depression Scale, patient category [defined as a dichotomous variable: PD (1) or control (0)], taking medicine for lowering cholesterol level [defined as a dichotomous variable: yes (1), no (0)], and APOE ε4 carrier [defined as a dichotomous variable: yes (1), no (0)]. Additional *post hoc* comparisons, quadratic correlation, and partial Pearson correlation within each group were performed, controlling for the abovementioned seven covariates. Similar results were observed with raw scores and without covariates.

In addition, we also investigated impacts of LDL, HDL, and TG on cognitive performance and GMv. Overall, the LDL results were highly similar to the main results with TC likely due to a strong correlation between LDL and TC in this sample (*r* = 0.87, *p* < 10^–36^, Supplementary Material). The results with LDL, HDL, and TG can be found in the Supplementary Material.

### Voxel-Based Morphometry Analysis

In the voxel-wise analysis, second-level multiple regression was used to test the effect of TC level on GMv, controlling for seven covariates listed above plus the TIV. The voxel and cluster thresholds were set at non-parametric *p* < 0.001 with TFCE (5000 permutations, and cluster-level *p* < 0.05 FWE-corrected) and 50 voxels, respectively (Smith and Nichols, 2009). GM threshold of 0.1 was used to ensure that voxels with less than 10% likelihood of GM were not included in this analysis.

In the ROI-based analysis, significant clusters from the voxel-wise analysis were identified as three ROIs. GMv of the three ROIs was calculated by adding all the voxels that have higher than 10% likelihood of being GM. Then, GMv were normalized by dividing the TIV, and times a constant 1000. Normalized GMv was then entered into the correlation analysis with SF score and the mediation analysis with SF score.

In addition, we further divided the 117 subjects into five groups based on TC levels [(100, 160), (160, 180), (180, 200), (200, 220), and (220, 300)]. In this way, the number of subjects was roughly the same across five groups (*n* = 25, 18, 26, 25, and 23, respectively; these five groups were not significantly different in terms of demographic variables). An analysis of covariance (ANCOVA) was performed to test whether the GMv in ROIs was different across these five groups.

## Data Availability Statement

The datasets are available to the public from the PPMI website.

## Ethics Statement

The studies involving human participants were reviewed and approved by PPMI. The patients/participants provided their written informed consent to participate in this study. Written informed consent was obtained from the individual(s) for the publication of any potentially identifiable images or data included in this article.

## Author Contributions

FY and MS analyzed the data. FY and XJ wrote the manuscript. FY, MS, and XJ revised the manuscript. All authors reviewed the manuscript.

## Conflict of Interest

The authors declare that this study received funding from PPMI. The primary funders were involved in the study design, collection, analysis, interpretation of data, the writing of this article, and the decision to submit it for publication. The following commercial companies co-fund PPMI and act in an advisory capacity: Abbvie, Avid Radiopharmaceuticals, Biogen Idec, Bristol-Myers Squibb, Covance, Eli Lilly & Co., F. Hoffman-La Roche, GE Healthcare, Genentech, GlaxoSmithKline, Lundbeck, Merck, MesoScale, Piramal, Pfizer, Prevail, and UCB.

## Footnotes

1 https://www.ppmi-info.org/

2 www.ppmi-info.org/

3 https://www.ppmi-info.org/study-design/

4 http://www.ppmi-info.org/wp-content/uploads/2017/06/PPMI-MRI-Operations-Manual-V7.pdf

5 www.fil.ion.ucl.ac.uk/spm/software/spm12/

6 www.neuro.uni-jena.de/cat/
