# Supplementary material for: Low Cholesterol Level Linked to Reduced Semantic Fluency Performance and Reduced Gray Matter Volume in the Medial Temporal Lobe
Source: Front Aging Neurosci. 2020 Mar 31;12:57. doi: 10.3389/fnagi.2020.00057 (PMC7142997; doi:10.3389/fnagi.2020.00057)
Supplement: Supplementary file 1 [file Data_Sheet_1.PDF]

Supplementary Materials

Low cholesterol level linked to reduced cognitive performance and reduced gray matter volume in the medial temporal lobe

Fan Nils Yang[1], Macdonell Stanford[1,2], Xiong Jiang[1]

[1]Department of neuroscience, [2] School of Medicine, Georgetown University Medical Center, Washington D.C., United State, 20057

Corresponding Author:

Xiong Jiang, PhD Department of

Neuroscience Georgetown University

Medical Center Washington, DC 20007

Phone: (1) 202-687-8928

Email: Xiong.Jiang@Georgetown.Edu

Results

LDL/HDL/TG

Similar analyses were performed using LDL/HDL/TG instead of TC as the independent variable. MANCOVA analysis revealed a significant effect between LDL levels and semantic fluency (SF) total scores ($F(1, 107) = 5.486$; $p = 0.021$), and between HDL levels and line orientation scores ($F(1, 107) = 4.783$; $p = 0.031$); but no significant effects between TG levels and any of the neuropsychological test scores (at least $p > 0.3$). Further quadratic regression analyses were performed between LDL levels and SF scores, and between HDL levels and line orientation scores, after adjusting for seven covariates: age, gender, education years, geriatric depression scale, patient category [defined as a dichotomous variable: PD (1) or control (0)], taking medicine for lowering cholesterol level [defined as a dichotomous variable: yes (1), no (0)], and APOE e4 carrier [defined as a dichotomous variable: yes (1), no (0)]. Results showed that a significant quadratic effect was found between LDL levels and adjusted SF total scores ($p < 0.005$, Supplementary Fig. 1), but not between HDL levels and line orientation scores ($p = 0.136$).

VBM analyses of GMv were conducted separately for the lTC and hTC groups, using the LDL as the regressor, after controlling for other potential confounding factors (see Methods). The VBM analyses revealed that no significant cluster was found in neither the lTC nor the hTC group. Similarly, no significant cluster was found when using HDL or TG as the regressor.

In addition, scatter plots of TC vs LDL, TC vs HDL, and TC vs TG, were shown in Supplementary Fig. 2. As there was a significant correlation between TC levels and HDL levels, we conducted additional analyses by adding HDL levels as a covariate, and obtained results that were similar to those included in the main article.

Control and PD Subjects

Similar to Fig. 2 in the main text, we examined the relationship between semantic fluency and TC in PD and Control participants, separately, and obtained similar results (see Supplementary Fig. 3).

Supplementary Figure 1. A quadratic relationship between adjusted semantic fluency (SF) test scores and LDL levels. The adjusted semantic fluency total scores were calculated by regressing out the covariates (age, gender, education years, geriatric depression scale, patient category, taking medicine for lowering cholesterol, and APOE ε4 carrier; see Methods). A significant quadratic correlation was found between adjusted SF and LDL.

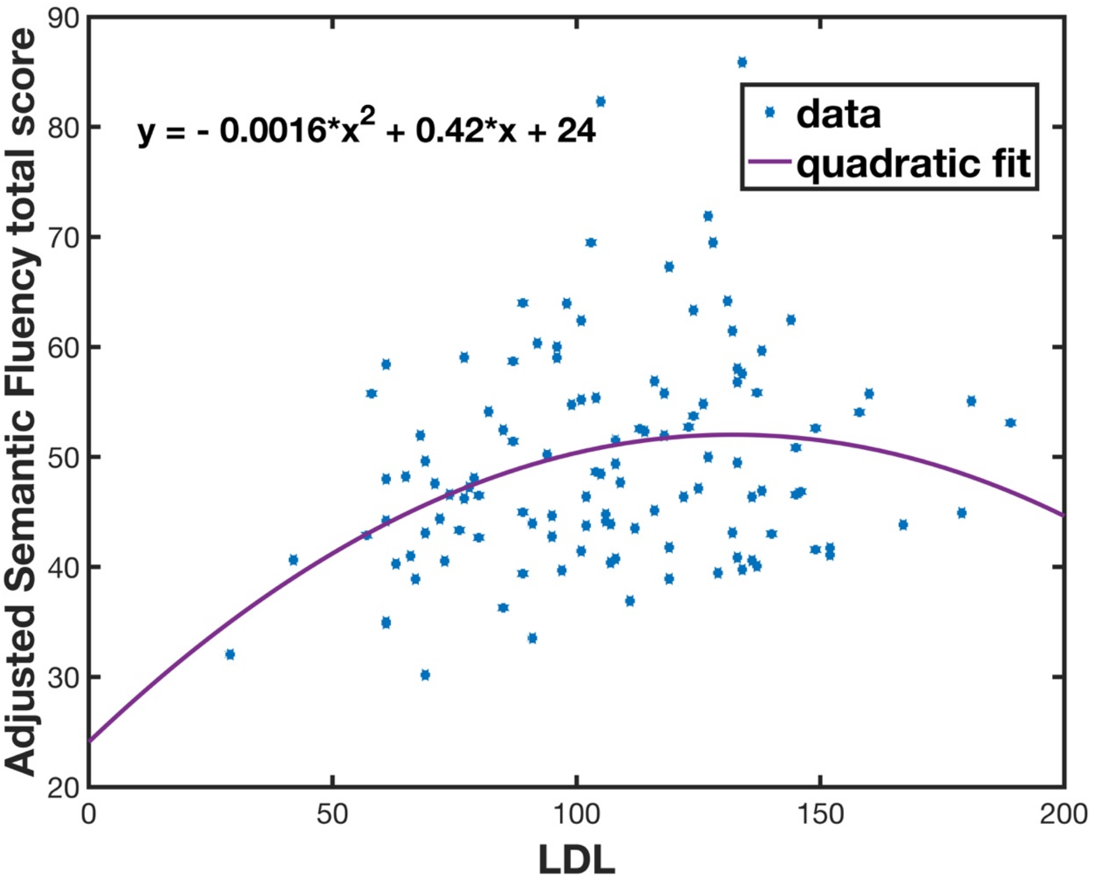

Supplementary Figure 2. Scatter plots of TC vs LDL, TC vs HDL, and TC vs TG in all participants. TC levels were significantly correlated with LDL (r = 0.873, $p < 10^{-36}$) and HDL (r = 0.374, p <0.0001) levels, but not TG levels (r = 0.015, p > 0.05).

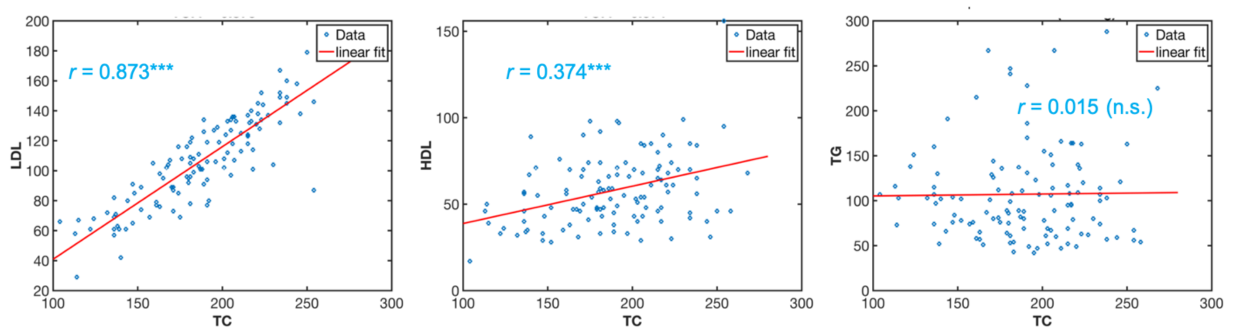

Supplementary Figure 3. The relationship between semantic fluency (SF) scores and total cholesterol (TC) in the lTC (left panel) and the hTC (right panel) group of Control (up panel) and PD (bottom panel) participants, separately. lTC group, TC < 200 mg/dl; hTC group, TC >= 200 mg/dl.

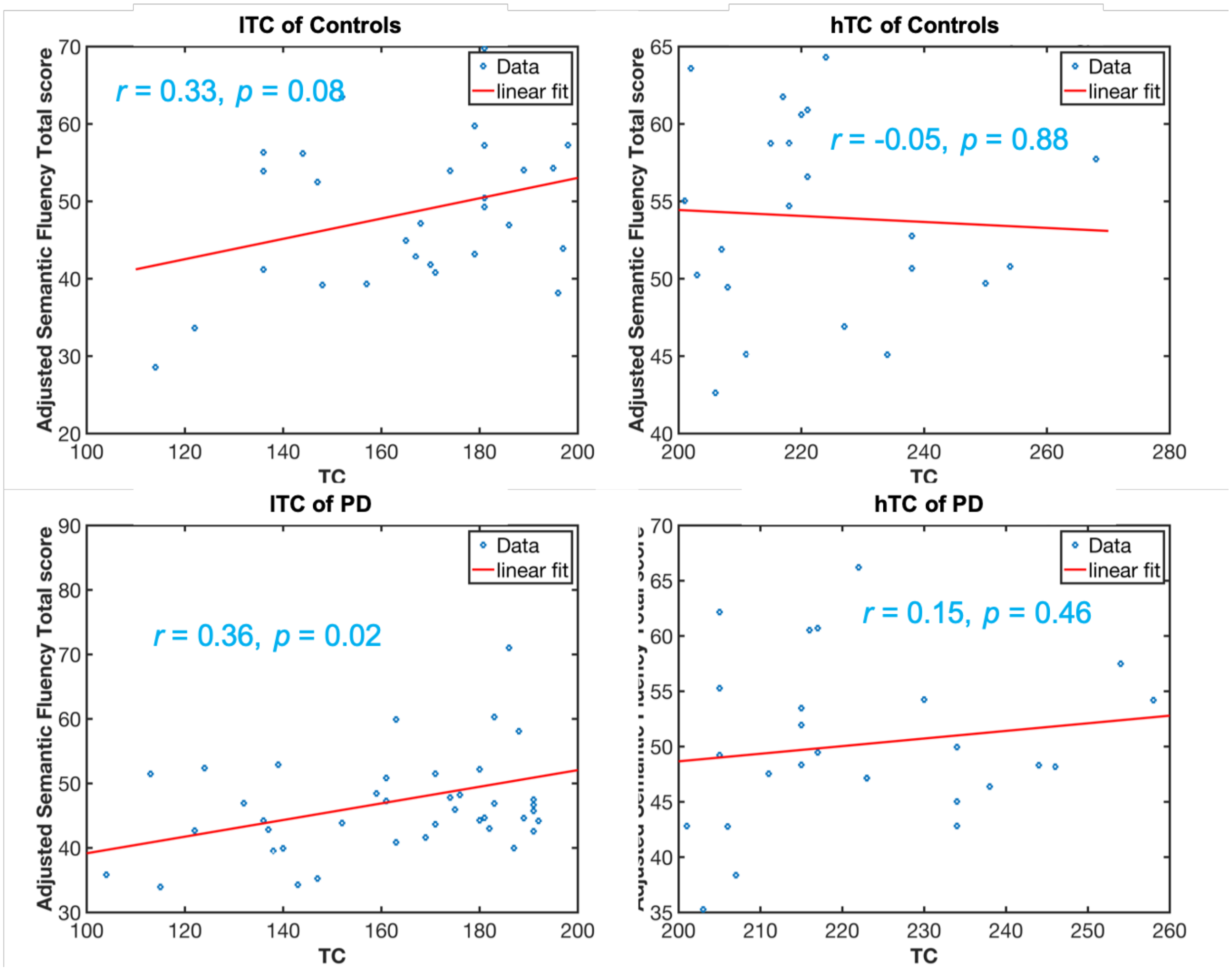

Supplementary Table 1. Demographic information of PD patients versus controls. Data are presented as mean (standard deviation).

Groups	PD (n=67)	Control (n=50)	Group Difference
Age	62.5 (9.7)	60.1 (8.9)	n.s.
Education	14.8 (3.0)	15.9 (2.7)	$p = 0.047$[b]
Gender (female%)[a]	37.3%	38.0%	n.s.
Disease duration (months)	7.3 (7.6)	N/A	N/A
Medicine (taking med %) [a]	32.8%	26.0%	n.s.
APOE ε4 carrier (%)[a]	32.8%	30%	n.s.
Geriatric Depression Scale	5.1 (1.2)	5.0 (1.0)	n.s.
Total Cholesterol	185.8 (36.8)	191.0 (35.3)	n.s.
Low Density Lipoprotein	102.9 (30.4)	112.7 (31.4)	n.s.
High Density Lipoprotein	58.6 (23.6)	56.8 (16.6)	n.s.
Triglycerides	103.2 (45.7)	112.4 (59.1)	n.s.

[a] Contingency χ^2 test.

[b] Uncorrected

PD, Parkinson's disease; n.s. not significant.

Supplementary Table 2. Neuropsychological test scores of PD patients versus controls. Data are presented as mean (standard deviation). The PD patients performed comparable to healthy controls, except on tests that are known to be affected in PD (including subtests in MoCA).

Cognitive domains	Tasks	lTC PD (n=41)	lTC Control (n=28)	hTC PD (n=26)	hTC Control (n=22)	*P* PD vs Controls	*P* lTC vs hTC	*P* interactions
Global	MoCA	27.1 (2.2)	28.3 (1.0)	26.8 (2.4)	28.5 (1.2)	0.001	n.s.	n.s.
Visuospatial	Line Orientation Score	12.8 (2.4)	13.6 (1.6)	13.0 (1.7)	13.1 (1.6)	n.s.	n.s.	n.s.
Executive abilities—working memory	Letter Number Sequencing Raw Score	10.8 (3.1)	10.5 (1.9)	10.3 (2.2)	11.3 (2.9)	n.s.	n.s.	n.s.
	Semantic Fluency Total Score	47.2 (11.5)	48.6 (11.2)	50.2 (10.2)	54.0 (9.6)	n.s.	n.s.	n.s.
Processing speed—attention	Symbol Digit Modalities Score	40.4 (11.3)	48.5 (9.6)	40.7 (8.6)	47.0 (12.4)	0.006	n.s.	n.s.
UPDRS III	PD rating	20.6 (8.2)	0.6 (1.0)	20.3 (8.1)	0.4 (1.0)	<0.001	n.s.	n.s.
Hoehn and Yahr Stage	Five stages PD progression	1.5 (0.5)	0.0 (0.0)	1.6 (0.5)	0.0 (0.0)	<0.001	n.s.	n.s.
Memory (HVLT-R)	Immediate Recall	24.8 (4.7)	25.2 (3.5)	24.3 (4.0)	26.4 (4.1)	n.s.	n.s.	n.s.
	Delayed Recall	8.0 (2.9)	8.8 (1.9)	8.1 (2.1)	9.2 (2.4)	n.s.	n.s.	n.s.
	Retention	0.8 (0.2)	0.9 (0.1)	0.8 (0.2)	0.9 (0.2)	n.s.	n.s.	n.s.
	Discrimination Recognition	10.0 (1.4)	9.6 (3.4)	9.9 (1.5)	9.2 (4.4)	n.s.	n.s.	n.s.

HVLT-R, Hopkins Verbal Learning Test - Revised; MoCA, the Montreal Cognitive Assessment; n.s., not significant; UPDRS, United Parkinson Disease Rating Scale.

Supplementary Table 3. Peak coordinates for significant correlation between low normal TC levels and reduced GMv in the lTC group

	Cluster Sizes	Coordinates		
Right ITL	59	40	33	-27
Right HIP/HPC	115	-21	-14	-16
Left HIP/HPC	154	-28	-36	-26

* ITL: inferior temporal lobe; HIP: hippocampus; HPC: parahippocampal cortex.
